# A phase 1/2, open-label assessment of the safety, tolerability, and efficacy of transdermal cannabidiol (ZYN002) for the treatment of pediatric fragile X syndrome

**DOI:** 10.1186/s11689-019-9277-x

**Published:** 2019-08-02

**Authors:** Helen Heussler, Jonathan Cohen, Natalie Silove, Nancy Tich, Marcel O. Bonn-Miller, Wei Du, Carol O’Neill, Terri Sebree

**Affiliations:** 1 Centre for Clinical Trials in Rare Neurodevelopmental Disorders, Children’s Health Queensland, Brisbane, Australia; 2 Centre for Child Health Research, University of Queensland, Brisbane, Australia; 3 Fragile X Alliance Inc. and Genetic Clinics Australia, Melbourne, Australia; 4 The Children’s Hospital at Westmead, Sydney, Australia; 5 Zynerba Pharmaceuticals, Devon, PA 19333 USA; 6 Clinical Statistics Consulting, Blue Bell, PA 19422 USA; 7 Canopy Growth Corporation, Smith Falls, Ontario V7A 0A8 Canada

**Keywords:** Cannabidiol, ZYN002, Zynerba, Transdermal, Fragile X, Pediatric

## Abstract

**Background:**

Fragile X syndrome (FXS) is characterized by a range of developmental, neuropsychiatric, and behavioral symptoms that cause significant impairment in those with the disorder. Cannabidiol (CBD) holds promise as a potential treatment for FXS symptoms due to its safety profile and positive effects on a number of emotional and behavioral symptoms associated with FXS. The aim of the current study was to evaluate the safety, tolerability, and initial efficacy of ZYN002, a transdermal CBD gel, in a pediatric population with FXS.

**Methods:**

Twenty children and adolescents (aged 6–17 years) with a diagnosis of FXS (confirmed through molecular documentation of FMR1 full mutation) were enrolled in an open-label, multi-site, trial of ZYN002. Transdermal CBD gel was administered twice daily for 12 weeks, titrated from 50 mg to a maximum daily dose of 250 mg. The primary efficacy endpoint was change from screening to week 12 on the Anxiety, Depression, and Mood Scale (ADAMS). Secondary endpoint measures included the Aberrant Behavior Checklist—Community for FXS (ABC-C_FXS_), Pediatric Anxiety Rating Scale (PARS-R), Pediatric Quality of Life Inventory (PedsQL™), three Visual Analogue Scales (VAS), and the Clinical Global Impression Scale—Severity (CGI-S) and Improvement (CGI-I).

**Results:**

The majority of treatment-emergent AEs (reported by 85% of participants) were mild in severity (70%), and no serious adverse events were reported. There was a statistically significant reduction in ADAMS total score from screening to week 12 and significant reductions on nearly all other secondary endpoints, including all ADAMS subscales (except depressed mood), all ABC-C_FXS_ subscale scores (e.g., social avoidance, irritability), PARS-R total severity score, and PedsQL total score.

**Conclusions:**

ZYN002 was well tolerated and produced clinically meaningful reductions in anxiety and behavioral symptoms in children and adolescents with FXS. These findings support further study of ZYN002 in a randomized, well-controlled trial for the treatment of behavioral symptoms of FXS.

**Trial registration:**

ANZCTR, ACTRN12617000150347 Registered 27 January 2017

## Introduction

Fragile X syndrome (FXS) is a rare genetic condition caused by cytosine-guanine-guanine (CGG) repeat expansion in the FMR1 gene located on the X chromosome [1]. CGG repeat expansion in the FMR1 gene, which silences the expression of the fragile X mental retardation protein (FMRP), is characteristically found in patients with FXS. FMRP, a ribonucleic acid (RNA) binding protein, is important for normal synaptic function, synaptic plasticity, and the development of neuronal connections during brain maturation. The absence of FMRP in neurons accounts for many of the neuropsychiatric symptoms of fragile X. Individuals with FXS exhibit a range of developmental and psychiatric symptoms, including anxiety (particularly social avoidance), hyperactivity, aggression, and negative affectivity/mood [2]. Anxiety and social avoidance are considered core features of FXS. One study found that 82.5% of the patients had at least one anxiety disorder, irrespective of sex, age, presence of autism, or IQ [3]. Social avoidance has been defined as a behavioral response to anxiety that arises from social interaction; thus, anxiety can be thought of as foundational precipitant to social avoidance. Social avoidance encompasses behaviors such as social isolation, social escape behaviors, and gaze avoidance that distance the individual from his/her social counterparts. Behavior problems concerning caregivers of children with FXS (*n* = 439) include anxiety, tantrums, and aggression while the participants also want drug treatment to reduce anxiety, address social anxiety, tantrums, and aggression [4]. Estimates of prevalence suggest that FXS affects approximately 1.4 in 10,000 males and 0.9 in 10,000 females, making it the most common heritable cause of intellectual disability [5].

Recent evidence suggests that dysregulation of the endocannabinoid system is central to the pathophysiology of FXS [6]. The endocannabinoid system is comprised of two G-protein-coupled receptors, cannabinoid receptor type 1 (CB_1_; located primarily in the central nervous system) and cannabinoid receptor type 2 (CB_2_; located in a number of systems throughout the body) [7, 8], as well as the endogenous cannabis-like ligands (endocannabinoids) anandamide (AEA) and 2-arachidonoylglycerol (2-AG). Both AEA and 2-AG bind to CB_1_ receptors and modulate synaptic transmission throughout the central nervous system [9, 10].

Cannabidiol (CBD), the primary non-euphoric cannabinoid contained in the *Cannabis sativa* L. plant, holds promise as a potential treatment for behavioral symptoms of FXS given its ability to interact with an FXS-compromised endocannabinoid system. Preclinical models have demonstrated that disruption of FMRP in FXS reduces the production of 2-AG, decreasing activation of CB_1_ receptors in the central nervous system [6]. Administration of CBD appears to increase 2-AG availability [11, 12], thereby increasing CB_1_ receptor activation and attenuating reduced endogenous cannabinoid signaling. Moreover, increased CB_1_ activation does not appear dependent on the presence of FMRP, as FMR1 knock out (KO) mice show no reduction in CB_1_ expression [13]. CBD administration also increases serum levels of the endogenous cannabinoid anandamide (AEA) [14], which could also produce modulating effects for FXS-related behavioral deficits. For example, higher baseline AEA among FMR1 KO mice is associated with greater cognitive performance [15], and administration of AEA in FMR1 KO mice reduces normally observed deficits in social behavior [16]. There is also evidence suggesting that CBD could promote improved synaptic plasticity by restoration of reduced neuronal long-term depression (LTD) [13], based on the metabolic glutamate receptor (mGluR) theory of FXS pathology ([17]). If substantiated, promotion of synaptic plasticity via CBD could lead to improvements in learning and cognition, a major deficit for patients with FXS. Finally, CBD is an allosteric modulator of GABA-A receptors [18]. Enhanced binding affinity for GABA could provide benefit to patients with FXS by reducing deleterious mood-related symptoms that are associated with low levels of GABA.

While a number of medications individually target some of the mechanisms reviewed above (e.g., GABA agonists), CBD has diffuse effects that could improve multiple symptoms experienced by patients with FXS. However, the majority of research on CBD as a therapeutic for psychological symptoms and associated behavior has not proceeded past the preclinical phase. Findings from the first cross-over trial testing CBD’s effects on symptoms of social anxiety in adults with social phobia found significant and clinically meaningful reductions in both physiological and cognitive indicators of anxiety [19]. This modulation of anxiety symptoms demonstrated in other clinical populations could translate to therapeutic effects for patients with FXS. A recent case series provided initial evidence that CBD may lead to broad improvement in childhood FXS symptomatology, including symptoms of anxiety and social avoidance [20]. CBD is also generally well tolerated in children and adults [21, 22], particularly when compared to many existing medications used to manage FXS.

Standard routes of CBD administration (e.g., oral, inhaled) may prove burdensome for use in pediatric populations and particularly those with neuropsychiatric and behavioral challenges. Transdermal application of a permeation-enhanced formulation of CBD may provide an easier, more convenient, and useful route of administration for parents/caregivers than oral CBD while still being an effective method of systemic delivery of cannabinoids [23]. An additional advantage of transdermal application is the bypass of first-pass metabolism, thus potentially providing a favorable pharmacokinetic profile.

The aims of the present study were to test the safety, tolerability, and efficacy of ZYN002, a transdermal, pharmaceutically manufactured, CBD gel, in a pediatric sample with FXS. We hypothesized that 12 weeks of ZYN002 treatment would be well-tolerated and lead to significant reductions in the most common and difficult behaviors associated with FXS.

## Methods

### Participants

Twenty male and female pediatric patients with FXS, 6 through 17 years of age at screening and a diagnosis of FXS confirmed through molecular documentation of FMR1 full mutation, were enrolled. For inclusion, patients were required to have a Pediatric Anxiety Rating Scale (PARS-R) [24] score ≥ 11, a Clinical Global Impression Severity [25] score ≥ 3, and be stable on their medication regimen for the 4 weeks preceding study enrollment and during the duration of the study. Patients were excluded if they suffered from any acute or progressive neurological disorder (progressive deterioration in functioning), psychosis, schizophrenia, psychiatric disorder, or severe mental abnormalities other than FXS, and/or if they had exposure to CBD or tetrahydrocannabinol (THC) in the 4 weeks preceding screening. Patient demographics appear in Table 1.

**Table 1:** Demographics for all treated patients as well as those completing treatment

	All treated patients (*N* = 20)	Patients completing week 12 (*N* = 18)
Age (years): mean (SD)	10.40 (3.88)	10.78 (3.86)
Range (min, max)	6–17	6–17
BMI (kg/m2): mean (SD)	19.34 (5.75)	19.50 (5.97)
Range (min, max)	12.60–35.00	12.60–35.00
Sex: frequency (%)		
Male	15 (75.00%)	14 (77.80%)
Female	5 (25.00%)	4 (22.20%)
Ethnicity: frequency (%)		
White, not Hispanic or Latino	18 (90.00%)	16 (88.90%)
Other (Middle Eastern)	2 (10.00%)	2 (11.10%)

### Procedure

Following parental consent, patients were screened for eligibility. Eligible patients were provided 12 weeks of ZYN002 as an adjunct to their existing treatments (6-week titration period, followed by 6-week maintenance period) at three possible dose levels: once daily 50 mg dose (dose A), twice daily 50 mg dose (100 mg total; dose B), or twice daily 125 mg dose (250 mg total; dose C). Dose increases from A to B and B to C were determined by assessment of tolerability; clinical response at weeks 2, 4, and 6 of the 6-week titration period; and investigator judgment. Maintenance dose was determined by the highest tolerated dose received during the titration period. At the end of the maintenance period, patients who had completed the first phase of the study were then provided the option of enrolling into a 24-month extension phase of the study. Patients not wishing to continue into the extension phase were either discontinued (if receiving dose A) or underwent a 1- or 2-week taper from study drug (doses B, C) if they were taking concomitant anti-epileptic drugs (AEDs). The current manuscript reports on the results of the initial 12-week study phase. The extension phase data collection is ongoing.

### Measures

Safety and tolerability were assessed biweekly through physical/neurological exam, vital sign collection, 12-lead electrocardiograms (ECGs), a Modified Suicidality Checklist, safety laboratory tests, pregnancy tests, urinalysis, and monitoring for adverse events (AEs).

Several measures assessing mood, behavior, and functioning were selected. The majority of measures were caregiver-reported, though a clinician-reported measure was also included to provide a multi-modal assessment of patient symptomatology across time.

#### Anxiety, Depression, and Mood Scale

The Anxiety, Depression, and Mood Scale (ADAMS) is a factor-derived measure of anxiety and mood symptoms [26] that has been validated in FXS samples [3]. Parents/caregivers are asked to rate the frequency and severity of 28 items on a scale of 0 “not a problem” to 3 “severe problem.” The ADAMS produces a total score and 5 subscale scores: manic/hyperactive behavior, depressed mood, social avoidance, general anxiety, and compulsive behavior.

#### Aberrant Behavior Checklist—Community for FXS

The Aberrant Behavior Checklist—Community for FXS (ABC-C_FXS_) is a 58-item scale used to measure parent/caregiver assessment of maladaptive behavior [27]. Factor analytic examination of the Aberrant Behavior Checklist—Community (ABC-C) within an FXS population yields a 6-factor solution [28] consisting of the following maladaptive behavior domains: social avoidance, stereotypic behavior, lethargy, irritability, hyperactivity, and inappropriate speech. Items are rated on a 4-point Likert scale ranging from 0 “not a problem at all” to 3 “the problem is severe in degree.”

#### Pediatric Anxiety Rating Scale

The Pediatric Anxiety Rating Scale (PARS-R) is a clinician-administered instrument that measures parent/caregiver assessments of 61 anxiety-related symptoms [24]. The PARS-R is validated for children with intellectual disability and is well correlated with parent-report and physician rating of anxiety [29]. Using a 5-point Likert scale, the interviewer assesses 7 anxiety domains, including number, frequency, and severity of anxiety symptoms; severity of physical symptoms of anxiety; avoidance of anxiety-provoking situations; and interference in family relationships and other relationships. For clinical trials, a total severity score is determined by summing 5 of the 7 domain scores. Higher scores reflect greater severity/impairment.

#### Pediatric Quality of Life Inventory

The Pediatric Quality of Life Inventory (PedsQL) asks parent/caregiver to rate 23 items related to core health dimensions assessed by the World Health Organization [30]. The scale produces a total score and subscale scores for physical, emotional, social, and school functioning, with good consistency and reliability [31].

#### Visual Analogue Scale

Parents/caregivers were asked to report on their child’s anxiety, hyperactivity, and tantrum/mood lability over the past week by marking on three 10-cm visual lines that were oriented between 0 “best behavior” and 10 “worst behavior.” The horizontal marks were measured in centimeters to quantify improvements or worsening of behavior since last assessment. Reliability for single-construct visual analogue scale (VAS) measures has been well established as proxies for more comprehensive measures of mood and anxiety [32].

#### Clinical Global Impression Scale—Severity (CGI-S) and Improvement (CGI-I)

The CGI was developed for clinicians in clinical trials to assess patients’ global functioning before and after intervention [33]*.* At baseline (i.e., screening), clinicians rated the severity of the patient’s current symptoms (CGI-S), while during the study, clinicians assessed how much the patient’s illness has improved or worsened relative to the baseline (i.e., screening) state (CGI-I) using two 7-point Likert-type scales. Lower scores on the CGI-S reflect better functioning, while lower scores on the CGI-I reflect greater improvements in symptoms. The CGI is well-validated and correlates with other standardized measures of psychiatric severity [34–36].

### Statistical Data Analysis Methods

Safety and tolerability were assessed by tabulation of the number and severity of AEs reported among all treated patients as well as monitoring for changes in physical and neurological exams, vital signs, laboratory values, pregnancy, modified suicidality checklist, and ECGs. Primary efficacy was assessed by testing for mean change from screening to week 12 in the total score and all subscale scores of the ADAMS. Secondary indicators of efficacy were assessed by testing for mean change from screening to week 12 in ABC-C_FXS_ subscale scores, PARS-R total severity score, PEDS-QL total and subscale scores, and mean VAS scores. Mean CGI-I scores (which assessed reported improvement at week 12) were summarized using descriptive statistics. All mean differences were tested with two-tailed paired-sample *t* tests (alpha = .05); nominal *p* values were reported as is without controlling for multiplicity. Given the small sample size, descriptive statistics of the median change and median percent change [Percent change = 100 × (change/screening)] were also calculated on each outcome measures’ distribution of change scores from screening to week 12. Analysis of efficacy included all patients who completed the 12 weeks of the trial.

## Results

A total of 20 patients were treated and included in the safety assessment. The majority of treated patients were male (75%), white (90%) with a mean age of 10.4 years. Half of the patients had co-morbid anxiety, one third had attention deficit hyperactivity disorder, and three patients had sleep disorders. Three patients had epilepsy, for which one patient was taking valproate 600 mg per day, one was taking valproate 100 mg twice daily, and one was taking lamotrigine 25 mg twice daily. No change in seizure frequency or antiepileptic drugs was reported during the study.

Retention in the study was excellent with 18 patients completing all 12 weeks of the trial and were included in efficacy analyses. Of the 18 patients who completed the study, 13 continued into the 24-month extension phase.

Of the 18 patients who completed 12 weeks of the trial, 16 (89%) received a daily maintenance dose of 250 mg (dose C) and 2 (11%) received a daily maintenance dose of 100 mg (dose B). One patient discontinued treatment on day 63 (100 mg daily; dose B) and 1 on day 64 (50 mg daily; dose A), 1 due to an AE of worsening of pre-existing eczema and the other, a sibling of the patient who withdrew due to the AE, withdrew consent for administrative reasons. Patients were titrated up to 250 mg/day at the investigator discretion based on ZYN002 tolerability and patient improvement based on the Clinical Global Impression—Improvement (CGI-I). Two patients were then down titrated to 100 mg. One patient was down titrated due to psychomotor hyperactivity and remained at 100 mg from week 8 through week 12. The other patient was down titrated at week 6 following an adverse event of stereotypy and remained at 100 mg for the remainder of the study.

### Safety and Tolerability

Frequencies of AEs are reported in Table 2. Seventeen of the 20 treated patients (85%) reported at least one treatment-emergent AE during the 12-week treatment phase. Six (30%) patients experienced at least one AE considered possibly or probably related to treatment, including two patients with application site disorders (mild dryness, moderate rash). Other AEs considered possibly related included symptoms of FXS (psychomotor hyperactivity, stereotypy, and nightmare). One patient discontinued treatment due to an AE (worsening of pre-existing eczema). The majority of AEs were mild in severity (70%) and resolved by the end of the 12-week treatment period with no dose adjustment. No serious adverse events (SAEs) were reported. AEs that were reported in 10% or more of the treated patients included gastroenteritis (25%), vomiting (10%), and upper respiratory tract infection (10%). No significant changes were observed in ECGs, physical/neurological exams, or vital signs (e.g., blood pressure, heart rate, respiratory rate). There were no clinically meaningful trends in laboratory values (including testosterone levels), except for an increase in eosinophil count at day 83 in the patient noted above who had a moderate rash, who completed the study. A repeat blood collection done 1 month later post-dose showed a slightly above normal eosinophil count. There were no clinically significant changes in liver function tests.

**Table 2:** Frequency of treatment-emergent adverse events (AEs)

All treated patients (*N* = 20)	
Treatment-emergent adverse events	Frequency (%)
Gastroenteritis	5 (25%)
Vomiting	2 (10%)
Upper respiratory tract infection	2 (10%)
Mouth ulceration	1 (5%)
Paraesthesia oral	1 (5%)
Diarrhea	1 (5%)
Application site dryness	1 (5%)
Application site rash	1 (5%)
Influenza	1 (5%)
Viral infection	1 (5%)
Viral upper respiratory tract infection	1 (5%)
Otitis media	1 (5%)
Tonsillitis	1 (5%)
Limb injury	1 (5%)
Eosinophil count abnormal	1 (5%)
Neck pain	1 (5%)
Pain in extremity	1 (5%)
Dizziness	1 (5%)
Lethargy	1 (5%)
Psychomotor hyperactivity	1 (5%)
Enuresis	1 (5%)
Stereotypy	1 (5%)
Nightmare	1 (5%)
Pruritus	1 (5%)
Eczema	1 (5%)
Rash pruritic	1 (5%)

Note. Frequency reflects all reported events; individual patients may have experienced more than one AE

### Primary Efficacy

**Fig. 1:**
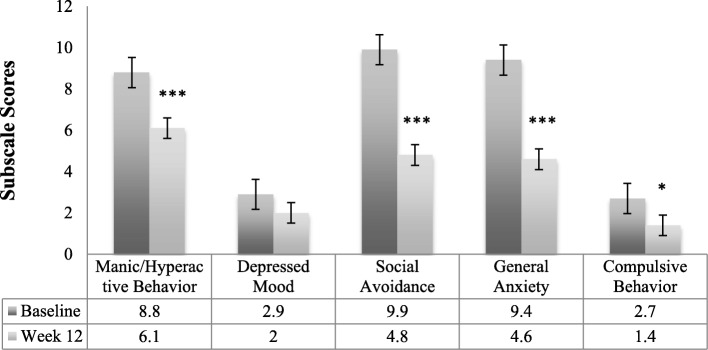
Change in mean ADAMS subscale scores from screening to week 12 (*N* = 18). *Note.* ADAMS = Anxiety, Depression and Mood Scale; Error bars represented standard error at each time point; * *p* < .05, **p < .01, ***p < .001

Results of the primary efficacy analysis for the ADAMS appear in Table 3, and results of analysis of the ADAMS subscales are represented in Fig. 1. There was a statistically significant reduction in the mean ADAMS total score (*t* = − 5.74, *p* < 0.001, *d* = 1.36) from screening to week 12. Patients also showed statistically significant mean reductions from screening to week 12 on the manic/hyperactive behavior (*t* = − 4.51, *p* < 0.001, *d* = 1.05), social avoidance (*t* = − 4.79, *p* < 0.001, *d* = 1.14), general anxiety (*t* = − 7.19, *p* < 0.001, *d* = 1.70), and compulsive behavior (*t* = − 2.43, *p* = 0.03, *d* = 0.56) subscales. Change from screening to week 12 on the depressed mood subscale failed to reach significance (*t* = − 1.54, *p* = 0.14, *d* = 0.37).

**Table 3:** Change in primary and secondary efficacy measures from screening to week 12

	Median score		Mean (SD) score		Median change from screening to week 12		
	Screening (*n* = 18)	Week 12 (*n* = 18)	Screening (*n* = 18)	Week 12 (*n* = 18)	Score (% change)	Paired-samples *t* test	
						*t*	Cohen’s *d*
Primary efficacy measures							
ADAMS							
Total	32.00	18.00	32.10 (14.36)	18.10 (8.32)	− 15.50 (− 48.90%)	− 5.74***	1.36
Manic/hyperactive	9.00	6.00	8.80 (3.99)	6.10 (3.29)	− 2.50 (− 32.50%)	− 4.51***	1.05
Depressed mood	2.00	1.00	2.90 (3.94)	2.00 (2.35)	0.00	− 1.54	0.37
Social avoidance	10.00	5.00	9.90 (5.18)	4.80 (2.07)	− 5.50 (− 55.40%)	− 4.79***	1.14
General anxiety	10.00	4.50	9.40 (4.35)	4.60 (3.35)	− 5.00 (− 52.30%)	− 7.19***	1.70
Compulsive behavior	2.50	1.50	2.70 (2.40)	1.40 (1.42)	− 2.00 (− 57.10%)	− 2.43*	0.56
Secondary efficacy measures							
ABC-C_FXS_							
Social avoidance	4.50	2.50	5.10 (3.46)	2.30 (2.22)	− 3.00 (− 66.70%)	− 4.31***	1.00
Stereotypy	8.50	2.00	8.10 (5.91)	3.20 (3.07)	− 5.00 (− 69.00%)	− 4.20***	0.99
Socially unresponsive/lethargic	8.00	4.00	9.20 (6.40)	4.10 (4.09)	− 5.00 (− 65.20%)	− 3.40**	0.76
Irritability	18.50	8.50	17.70 (12.68)	10.60 (11.03)	− 7.50 (− 50.00%)	− 2.92**	0.69
Hyperactivity	10.50	8.50	13.70 (9.09)	9.80 (7.38)	− 3.50 (− 29.80%)	− 2.49*	0.59
Inappropriate speech	6.00	3.50	5.90 (2.30)	3.50 (2.66)	− 3.00 (− 44.40%)	− 3.69**	0.87
PARS-R							
Total severity (5-item)	14.50	9.50	15.70 (3.88)	10.60 (3.43)	− 6.00 (− 38.20%)	− 4.18***	0.98
PedsQL							
Total score	65.20	67.80	57.80 (18.78)	67.70 (18.27)	9.80 (16.70%)	2.98*	0.77
Physical functioning	68.80	79.70	67.90 (27.36)	78.00 (22.39)	6.30 (7.10%)	2.04	0.53
Emotional functioning	60.00	80.00	64.00 (20.72)	78.30 (16.63)	10.00 (11.10%)	2.27*	0.58
Social functioning	40.00	50.00	37.30 (24.70)	49.00 (24.35)	10.00 (27.80%)	1.95	0.50
School functioning	55.00	55.00	55.70 (19.17)	59.10 (22.47)	0.00	0.95	0.25
Psychosocial health	51.70	62.90	52.40 (17.22)	62.20 (18.91)	8.30 (16.10%)	2.25*	0.58
VAS							
Hyperactivity/impulsivity	6.70	3.50	5.90 (2.43)	3.60 (2.49)	− 1.70 (− 44.60%)	− 4.77***	1.10
Tantrum/mood lability	5.40	2.90	4.70 (2.09)	3.20 (2.18)	− 1.00 (− 33.90%)	− 3.59**	0.86
Anxiety	6.50	3.80	6.00 (2.05)	3.80 (1.93)	− 2.20 (− 35.40%)	− 4.25***	1.01
CGI							
CGI-S	5.00	–	5.10 (1.39)	–	–	–	–
CGI-I	–	2.00	–	2.50 (1.01)	–	–	–

Note. Prior to tabulation change in score at week 12 was calculated for each subject by (change = score at week 12 − score at screening), and percent change in score at week 12 was calculated for each subject by [% Change = 100 × (change at week 12/score at screening)]

*p<.05 compared to screening

**p<.01 compared to screening

***p<.001 compared to screening

### Secondary Efficacy

Consistent with the results of the primary efficacy analysis, patients taking ZYN002 demonstrated statistically and clinically significant 12-week reductions in all subscales of the ABC-C_FXS_ [i.e., social avoidance (*t* = − 4.31, *p* < 0.001, *d* = 1.00), stereotypy (*t* = − 4.20, *p* < 0.001, *d* = 0.99), socially unresponsive/lethargic (*t* = − 3.40, *p* < 0.01, *d* = 0.76), irritability (*t* = − 2.92, *p* < 0.01, *d* = 0.69), hyperactivity (*t* = − 2.48, *p* = 0.02, *d* = 0.58), and inappropriate speech (*t* = − 3.69, *p* < 0.01, *d* = 0.87)]. Patients also showed significant improvements between screening and week 12 on the PARS-R (*t* = − 4.18, *p* < 0.001, *d* = 0.98), the PedsQL total score (*t* = 2.98, *p* < 0.05, *d* = 0.77), and the subscale scores for psychosocial health (*t* = 2.25, *p* < 0.05, *d* = 0.58) and emotional functioning (*t* = 2.27, *p* < 0.05, *d* = 0.58). Likewise, VAS measures of hyperactivity/impulsivity (*t* = − 4.77, *p* < 0.001, *d* = 1.10), tantrum/mood lability (*t* = − 3.59, *p* < 0.01, *d* = 0.86), and anxiety (*t* = − 4.25, *p* < 0.001, *d* = 1.01) all showed significant improvement. Mean CGI-S at screening was 5.10 (SD = 1.39), indicating “marked” impairment in functioning at baseline, while the mean CGI-I at week 12 was 2.50 (SD = 1.01), indicating “minimally” to “much” improved symptomatology following treatment. The only secondary measures of mean change that failed to reach significance were the PedsQL physical functioning (*t* = 2.04, *p* = 0.06, *d* = 0.53), school functioning (*t* = .95, *p* = .36, *d* = 0.25), and social functioning (*t* = 1.95, *p* = 0.07, *d* = 0.50) subscales. Results of all secondary measures are summarized in Table 3.

## Discussion

In this open-label study, ZYN002 CBD gel was well tolerated and produced clinically and statistically meaningful reductions in anxiety and behavioral symptoms among children and adolescents with FXS. Eighteen (90.0%) of the 20 enrolled patients completed 12 weeks of treatment, and most patients received the maximum planned dose of 250 mg/day during weeks 6 to 12 of treatment (i.e., the maintenance period). The majority of reported AEs were mild in severity and resolved by the end of the 12-week treatment period. There were no SAEs reported. These results suggest clinical safety and tolerability for the treatment of FXS patients with ZYN002 gel.

Results from the efficacy analyses all converge to suggest a pattern of clinical improvement in a range of key parent-rated emotional and behavioral symptoms of FXS. Patients in this open-label trial saw significant 12-week improvement over screening scores for the majority of study efficacy endpoints, including the primary endpoint of change from screening to week 12 in the ADAMS total score and 4 out of the 5 ADAMS subscale scores, as well as the secondary 12-week endpoints of ABC-C_FXS_, PARS-R, PedsQL, VAS, and CGI-I. The greatest emotional improvement following treatment was observed for anxiety (ADAMS, PARS-R, VAS; *d* = 0.98 to 1.70), while behavioral improvements were most pronounced in the domains of social avoidance (ABC-C_FXS_; *d* = 1.00) and stereotypy (ABC-C_FXS_; *d* = 0.99). Importantly, observed improvements were generally greater than those demonstrated for placebo in prior controlled clinical trials in FXS [37, 38]. Across assessments, there was consensus in improvement in both internalizing (e.g., anxiety, social avoidance) and externalizing symptoms (e.g., irritability) over the course of treatment. Consistent with observations of change in the ADAMS and ABC-C_FXS_ domains, patients experienced the greatest functional improvements in domains specifically related to emotional and psychosocial functioning.

The current study’s findings are limited by the open-label design and small sample size. Without a placebo control, the true effects of a drug cannot be determined. The majority of patients showed improvement on most outcome variables, with these effects being large and generally not dependent on sex, age, or body mass index (BMI). Even those secondary endpoints that failed to reach statistical significance were still associated with moderate effect sizes (*d* = 0.25 to 0.53). Moreover, results from the current study are consistent with data from previous trials of CBD for overlapping behavioral/mood disorders [19], extending those findings to a clinical population of children with FXS.

As no medications are approved specifically for the treatment of FXS, and recent drug development efforts have been generally unsuccessful [39], the present safety and efficacy data combine to suggest that a well-controlled, randomized trial testing ZYN002 CBD gel for the treatment of behavioral symptoms of FXS is paramount.

## Conclusions

Results from this open-label trial indicate that ZYN002 may be an effective treatment for many behavioral and emotional symptoms associated with FXS, while theory, preclinical data, and early case reports have pointed toward the therapeutic potential of CBD for the treatment of FXS. Given the lack of medications approved for the treatment of FXS, this open-label study findings highlight the urgent need for randomized, controlled, clinical trials to further assess the safety and efficacy of ZYN002 for FXS symptoms ranging from social avoidance, irritability, social unresponsiveness/lethargy, and stereotypy, to anxiety.

## Data Availability

The datasets generated and/or analyzed during the current study are not publically available for reasons of confidentiality of patient and intellectual property information.

## Abbreviations

2-AG: 2-Arachidonoylglycerol
ABC-C: Aberrant Behavior Checklist—Community
ABC-C_FXS_: Aberrant Behavior Checklist—Community for FXS
ADAMS: Anxiety, Depression, and Mood Scale
AEA: Anandamide
AEDs: Anti-epileptic drugs
AEs: Adverse events
BMI: Body mass index
CB_1_: Cannabinoid receptor type 1
CB_2_: Cannabinoid receptor type 2
CBD: Cannabidiol
CGG: Cytosine-guanine-guanine
CGI-I: Clinical Global Impression Scale—Improvement
CGI-S: Clinical Global Impression Scale—Severity
ECGs: 12-lead electrocardiograms
FMR1: Fragile X mental retardation 1
FMRP: Fragile X mental retardation protein
FXS: Fragile X syndrome
GABA: Gamma-aminobutyric acid
KO: Knock out
LTD: Long-term depression
Mg: Milligrams
mGluR: Metabolic glutamate receptor
PARS-R: Pediatric Anxiety Rating Scale
PedsQL™: Pediatric Quality of Life Inventory
RNA: Ribonucleic acid
SAE: Serious adverse event
THC: Tetrahydrocannabinol
VAS: Visual analogue scale
